# Numerical Study on Convective Heat Transfer Enhancement in Horizontal Rectangle Enclosures Filled with Ag-Ga Nanofluid

**DOI:** 10.1186/s11671-017-2095-8

**Published:** 2017-05-04

**Authors:** Cong Qi, Liyuan Yang, Guiqing Wang

**Affiliations:** School of Electrical and Power Engineering, China University of Mining and Technology, Xuzhou, 221116 China

**Keywords:** Nanofluid, Natural convection, Heat transfer enhancement, Two-phase lattice Boltzmann method

## Abstract

The natural convection heat transfer of horizontal rectangle enclosures with different aspect ratios (*A* = 2:1 and *A* = 4:1) filled with Ag-Ga nanofluid (different nanoparticle volume fractions *φ* = 0.01, *φ* = 0.03, *φ* = 0.05 and radiuses *r* = 20 nm, *r* = 40 nm, *r* = 80 nm) at different Rayleigh numbers (*Ra* = 1 × 10^3^ and *Ra* = 1 × 10^5^) is investigated by a two-phase lattice Boltzmann model. It is found that the Nusselt number enhancement ratios of two enclosures (*A* = 2:1 and *A* = 4:1) filled with Ag-Ga nanofluid (*r* = 20 nm) are the same compared with those of the water at the corresponding aspect ratio enclosure. The more flat horizontal rectangular enclosure (*A* = 4:1) has the higher Nusselt number than the less flat horizontal rectangular enclosure (*A* = 2:1). It is also found that Nusselt number increases with the decreasing nanoparticle radius. Nusselt number enhancement ratios for every nanoparticle radius reducing by half at high Rayleigh number are higher than those at low Rayleigh number in most cases. The interaction forces between particles are also investigated in this paper. It is found that the Brownian force *F*_B_ is about two magnitudes greater than that of drag force *F*_D_, and the value of driving force *F*_S_ in *A* = 4:1 enclosure is about twice the value of driving force *F*_S_ in *A* = 2:1 enclosure while other forces are almost the same.

## Background

Heat transfer enhancement attracts more and more people’s attention. One method is to improve the structure of the heat exchanger, and another method is to find new fluid with higher heat transfer performance instead of the common fluid. People have studied the structures of the heat exchangers for many years. About the heat transfer medium, since the nanofluid with high thermal conductivity is prepared, the thermal properties [1–3] and heat transfer performance [4–9] of nanofluid are studied by more and more researchers.

Natural convection heat transfer is an important heat transfer process. The natural convection heat transfer characteristics of nanofluid have been widely investigated by experimental and numerical methods respectively.

Natural convection heat transfer characteristics of nanofluid are experimentally investigated by many researchers. Ho et al. [10] experimentally investigated the natural convection heat transfer of Al_2_O_3_-water nanofluid in different size enclosures respectively, and the effects of nanoparticle volume fraction and Rayleigh number on the natural convection heat transfer of nanofluid are discussed. Heris et al. [11–13] experimentally investigated the laminar flow convective heat transfer of CuO-water, Al_2_O_3_-water, and Cu-water nanofluid in a circular tube respectively. Hu et al. [14] experimentally investigated the natural convection heat transfer of TiO_2_-water nanofluid with different nanoparticle mass fractions, and the effects of Rayleigh number on natural convection heat transfer are discussed. Sommers et al. [15] experimentally investigated the convection heat transfer of Al_2_O_3_-propanol nanofluid through a copper pipe, and the effects of heat rate on the convection heat transfer are discussed.

In addition to the experimental method, numerical simulation is also an important method to study the natural convection heat transfer of nanofluid. Many researchers have investigated the natural convection heat transfer of nanofluid by various numerical methods. He et al. [16] investigated the convection heat transfer of TiO_2_-water nanofluid flowing through a straight tube by a single-phase method and a combined Euler and Lagrange method respectively. The effects of nanoparticle fraction, Reynolds number, and nanoparticle aggregated size on the convection heat transfer are discussed. Bianco et al. [17–19] investigated the convection flow of a circular tube filled with Al_2_O_3_-water nanofluid under different conditions respectively. Akbarinia et al. [20–22] investigated the mixed convection of Al_2_O_3_-water nanofluid in a horizontal curved tube, annulus and elliptic ducts respectively. Sheikholeslami et al. [23–27] investigated the natural convection heat transfer of various kinds of nanofluid under magnetic field and revealed the heat transfer enhancement mechanism of nanofluid. Qi et al. [28–30] investigated the natural convection heat transfer of Cu/Al_2_O_3_-water, Al_2_O_3_-water, and Cu-gallium in an enclosure by a lattice Boltzmann method respectively.

The above literatures made a great contribution to the researches on the effects of macro-factors (nanoparticle volume fraction, kinds of nanofluid, and so on) on the heat transfer of nanofluid. The effects of micro-factors (nanoparticle radius) on the heat transfer of nanofluid are needed to be studied. Hence, in our previous published paper [31], the natural convection heat transfer of a vertical rectangle enclosure (the left and the right walls are hot and cold walls, respectively, and other walls are adiabatic) filled with Cu-Ga nanofluid with various radius nanoparticles is investigated. In order to reveal the effects of different laying forms of the rectangle enclosure and different boundary conditions in the natural convection heat transfer of nanofluid with different nanoparticle radiuses, the natural convection of a horizontal rectangle enclosure (the left and right walls are all hot walls, and other walls are all cold walls) filled with Ag-Ga nanofluid with various radius nanoparticles is investigated in this paper.

## Methods

The natural convection of the horizontal rectangle enclosure filled with Ag-Ga nanofluid with various radiuses of nanoparticles is simulated by a two-phase lattice Boltzmann model. The two-phase lattice Boltzmann model for nanofluid has been developed by us in the previous published paper [31]. The main basic equations of the two-phase lattice Boltzmann model are given as follows:

The evolution equations and equilibrium distribution functions for velocity and temperature fields are given respectively as follows:

1 $$ {f}_{\alpha}^{\sigma}\left(\boldsymbol{r}+{\boldsymbol{e}}_{\alpha}{\delta}_t, t+{\delta}_t\right)-{f}_{\alpha}^{\sigma}\left(\boldsymbol{r}, t\right)=-\frac{1}{\tau_f^{\sigma}}\left[{f}_{\alpha}^{\sigma}\left(\boldsymbol{r}, t\right)-{f}_{\alpha}^{\sigma eq}\left(\boldsymbol{r}, t\right)\right]+\frac{2{\tau}_f^{\sigma}-1}{2{\tau}_f^{\sigma}}\cdot \frac{F_{\alpha}^{\sigma}{\delta}_t{\boldsymbol{e}}_{\alpha}}{B_{\alpha}{c}^2}+{\delta}_t{F}_{\alpha}^{\sigma \hbox{'}} $$

2 $$ {f}_{\alpha}^{\sigma \mathrm{eq}}={\rho}^{\sigma}{w}_{\alpha}\left[1+\frac{{\boldsymbol{e}}_{\alpha}\cdot {\boldsymbol{u}}^{\sigma}}{c_{\mathrm{s}}^2}+\frac{{\left({\boldsymbol{e}}_{\alpha}\cdot {\boldsymbol{u}}^{\sigma}\right)}^2}{2{c}_{\mathrm{s}}^4}-\frac{{u^{\sigma}}^2}{2{c}_{\mathrm{s}}^2}\right] $$

3 $$ {T}_{\alpha}^{\sigma}\left(\boldsymbol{r}+{\boldsymbol{e}}_{\alpha}{\delta}_t, t+{\delta}_t\right)-{T}_{\alpha}^{\sigma}\left(\boldsymbol{r}, t\right)=-\frac{1}{\tau_T^{\sigma}}\left[{T}_{\alpha}^{\sigma}\left(\boldsymbol{r}, t\right)-{T}_{\alpha}^{\sigma \mathrm{eq}}\left(\boldsymbol{r}, t\right)\right] $$

4 $$ {T}_{\alpha}^{\sigma \mathrm{eq}}={w}_a{T}^{\sigma}\left[1+3\frac{{\boldsymbol{e}}_{\alpha}\cdot {\boldsymbol{u}}^{\sigma}}{c^2}+4.5\frac{{\left({\boldsymbol{e}}_{\alpha}\cdot {\boldsymbol{u}}^{\sigma}\right)}^2}{2{c}^4}-1.5\frac{{u^{\sigma}}^2}{2{c}^2}\right] $$

The interaction forces including gravity and buoyancy force *F*_H_, drag force *F*_D_, interaction potential force *F*_A_, and Brownian force ***F***_B_ are presented respectively as follows:

5 $$ {\boldsymbol{F}}_{\mathrm{H}}=-\frac{4\pi {a}^3}{3} g\varDelta {\rho}^{\hbox{'}} $$

6 $$ {\boldsymbol{F}}_{\mathrm{D}}=-6\pi \mu a\varDelta u $$

7 $$ {\boldsymbol{F}}_{\mathrm{A}}={\displaystyle \sum_{i=1}^8{n}_i}\frac{\partial {V}_{\mathrm{A}}}{\partial {\boldsymbol{r}}_i} $$

8 $$ {\boldsymbol{F}}_{\mathrm{B}}={G}_i\sqrt{\frac{C}{dt}} $$

All units in the simulation adopt the lattice units. The transformation relationships between lattice units and international units are as follows:

9 $$ \left\{\begin{array}{l}{t}^{\hbox{'}}=\frac{t}{T},\kern0.5em {l}^{\hbox{'}}=\frac{l}{L},\kern0.5em {u}^{\hbox{'}}= u\frac{T}{L}= u\frac{\nu^{\hbox{'}}}{\nu}\frac{D}{D^{\hbox{'}}},\kern0.5em {a}^{\hbox{'}}= a\frac{T^2}{L}= a{\left(\frac{\nu^{\hbox{'}}}{\nu}\right)}^2{\left(\frac{D}{D^{\hbox{'}}}\right)}^3\\ {}{m}^{\hbox{'}}=\frac{m}{G}= m\frac{\rho^{\hbox{'}}}{\rho}{\left(\frac{D^{\hbox{'}}}{D}\right)}^3,\kern0.5em {F}^{\hbox{'}}= F\frac{T^2}{G L}= F\frac{\rho^{\hbox{'}}}{\rho}{\left(\frac{\nu^{\hbox{'}}}{\nu}\right)}^2\end{array}\right. $$

where the physical quantities with the superscript “^'^” represent the lattice units and the physical quantities without the superscript “^'^” represent the international units.

The other details of this model can be seen in the previous published paper [31].

## Results and Discussion

**Fig. 1:**
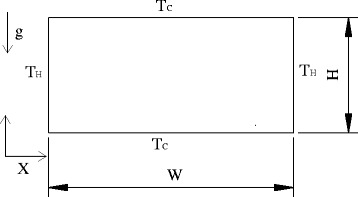
Rectangular enclosure. The left and right walls of the rectangular enclosure keep a high temperature *T*_H_, and the top and bottom walls keep a low temperature *T*_C_. The width and height of the rectangular enclosure are *W* and *H* respectively

Figure 1 presents the horizontal rectangular enclosure filled with Ag-Ga nanofluid in the simulation. The aspect ratio between width and height is defined as *A* = *W*/*H*. The thermo-physical parameters of liquid metal gallium (Ga) and silver (Ag) nanoparticles are given in Table 1. Because the temperature change has a great effect on the specific heat of Ga while a mall effect on the specific heat of Ag, in order to simplify the calculation, the specific heat of Ga considers the effect of temperature, and the specific heat of Ag keeps a constant. The left and right walls of the horizontal rectangular enclosure are all hot wall *T*_H_, and the top and bottom walls are all cold wall *T*_C_. The initialization conditions of the four walls are shown as follows:

**Table 1:** Thermo-physical parameters. Thermo-physical parameters of liquid metal gallium and silver nanoparticle

Physical properties	Base fluid (Ga) [30]	Nanoparticle (Ag) [32]
*ρ*(kg/m^3^)	6090	10,500
*c*_p_(J/kg k)	429.9–0.275543 T	235
*μ*(m^2^/s)	0.0018879	/
*k*(W m^−1^ K^−1^)	31	429

10 $$ \left\{\begin{array}{l} x=0\kern2em \boldsymbol{u}=0,\kern1em  T=1;\kern2.5em  x=1\kern2em \boldsymbol{u}=0,\kern1em  T=1\ \\ {} y=0\kern1.5em \boldsymbol{u}=0,\kern1em  T=0;\kern3em  y=1\kern1.5em \boldsymbol{u}=0,\kern1em  T=0\end{array}\right. $$

Before the study in Ag-Ga nanofluid, a grid independence test is analyzed. Five kinds of grids (78 × 39, 128 × 64, 198 × 99, 256 × 128, and 300 × 150) are chosen to be tested in this paper. The results under different grids are showed in Table 2. It can be seen from Table 2 that there are noticeable differences in the results from 78 × 39 to 256 × 128 but few differences from 256 × 128 to 300 × 150. In order to accelerate the numerical simulation velocity, the grid 256 × 128 is adopted for *A* = 2:1 enclosure in this numerical simulation. Correspondingly, the grid 256 × 64 is adopted for *A* = 4:1 enclosure.

**Table 2:** Grid independence test. Numerical simulation results at different grids (*Ra* = 1 × 10^5^, *φ* = 0.05)

Grid number	78 × 39	128 × 64	198 × 99	256 × 128	300 × 150
*Nu*_avg_	1.583	1.770	1.789	1.805	1.806

The reliability and accuracy of the two-phase lattice Boltzmann model have been verified in the previous published paper [31].

**Fig. 2:**
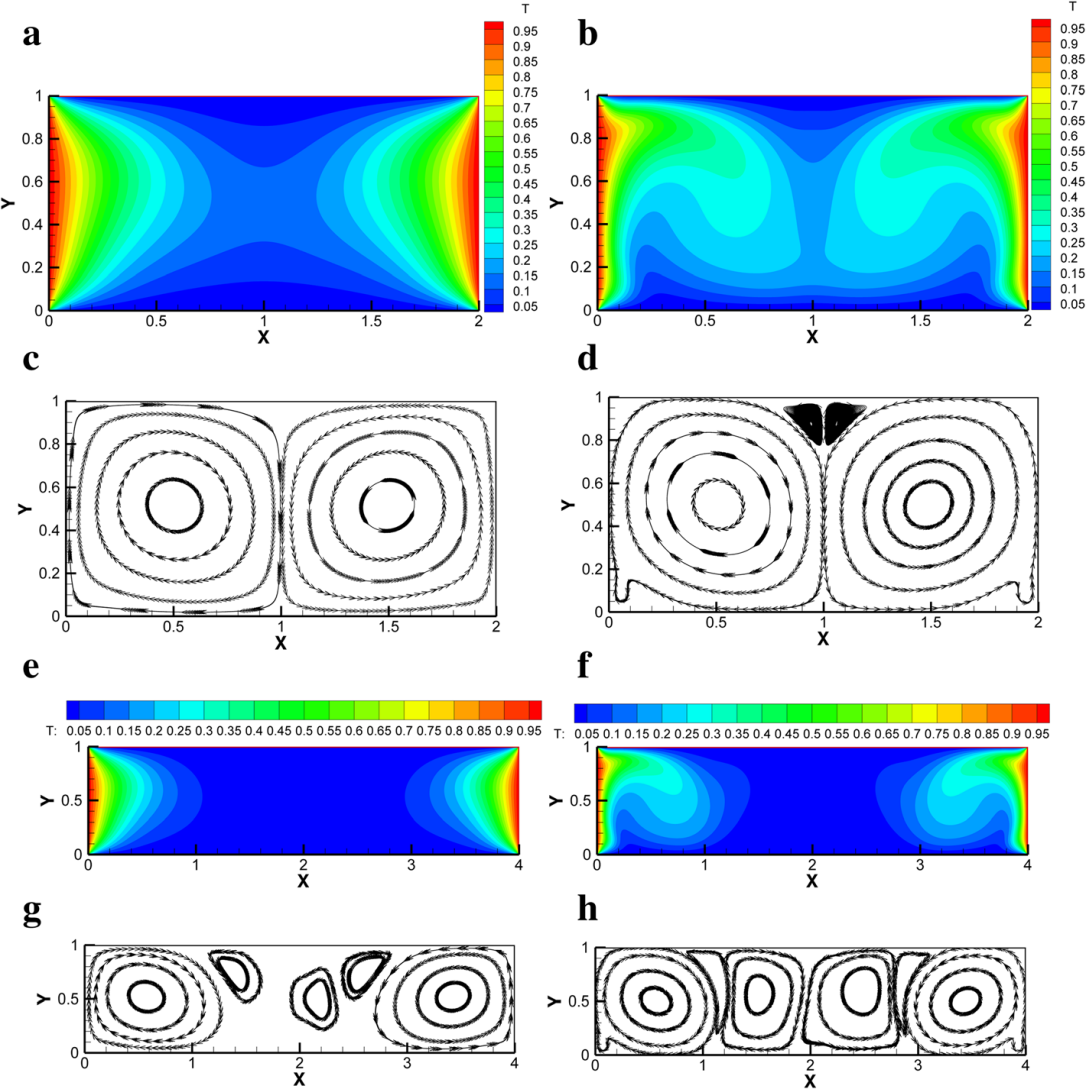
Temperature nephogram and streamlines of Ag-Ga nanofluid. Temperature nephogram and streamlines of Ag-Ga nanofluid (*r* = 20 nm, *φ* = 0.01) in the horizontal rectangular enclosure (*A* = 2:1 and *A* = 4:1). Temperature nephogram: **a** *Ra* = 1 × 10^3^, *A* = 2:1. **b** *Ra* = 1 × 10^5^, *A* = 2:1. **e** *Ra* = 1 × 10^3^, *A* = 4:1. **f** *Ra* = 1 × 10^5^, *A* = 4:1. Streamlines: **c** *Ra* = 1 × 10^3^, *A* = 2:1. **d** *Ra* = 1 × 10^5^, *A* = 2:1. **g** *Ra* = 1 × 10^3^, *A* = 4:1. **h** *Ra* = 1 × 10^5^, *A* = 4:1

Figure 2 shows the temperature nephogram and streamlines of Ag-Ga nanofluid (*r* = 20 nm, *φ* = 0.01) in the horizontal rectangular enclosure (*A* = 2:1 and *A* = 4:1) at different Rayleigh numbers (*Ra* = 1 × 10^3^ and *Ra* = 1 × 10^5^) respectively. It can be seen that the isotherm becomes more and more crooked with the Rayleigh number. The number of vortexes in the enclosure increases with the Rayleigh number. The vortexes can disturb the laminar boundary layer and enhance the heat transfer. The main heat transfer form is heat conduction at low Rayleigh number *Ra* = 1 × 10^3^, while the main heat transfer form changes from heat conduction to convection heat transfer at high Rayleigh number *Ra* = 1 × 10^5^. High Rayleigh number causes a big temperature difference driving force which disturbs the laminar boundary layer and improves the heat transfer compared with the low Rayleigh number.

**Fig. 3:**
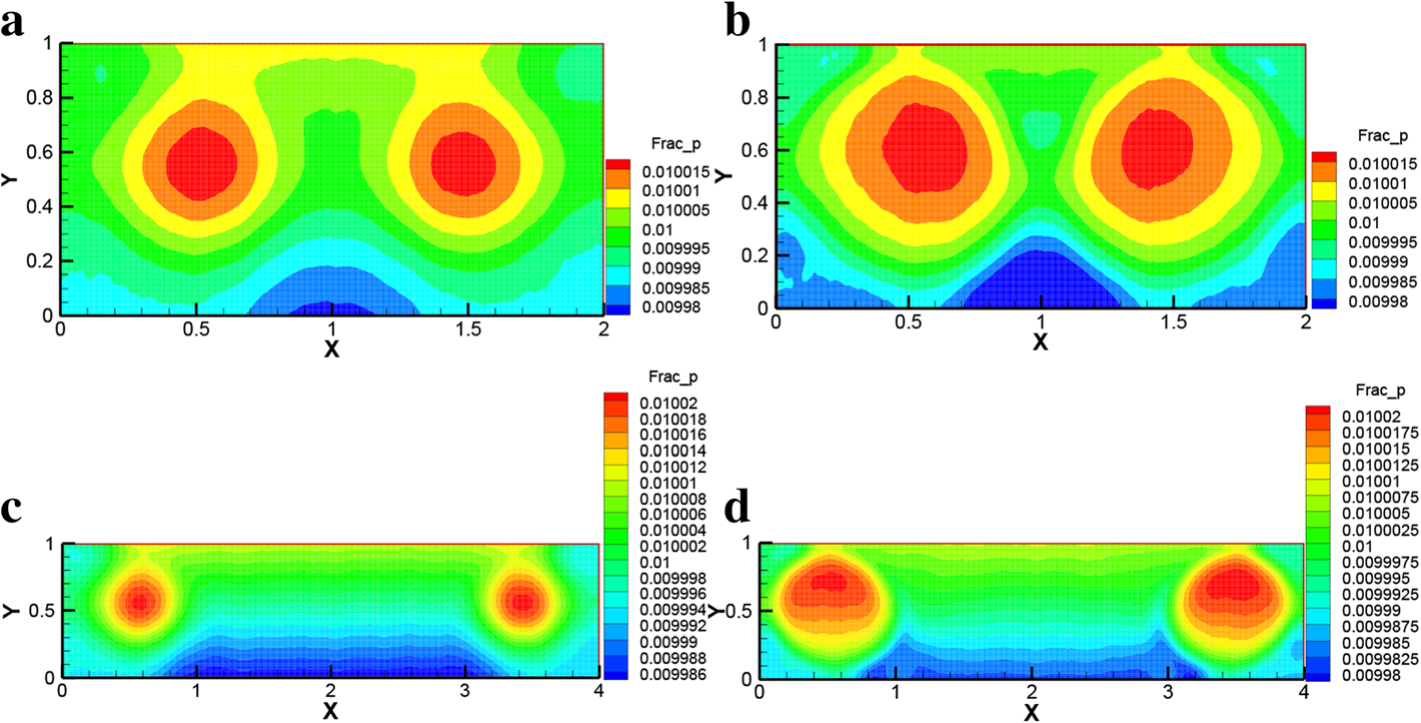
Nanoparticle volume fraction distributions of Ag-Ga nanofluid. Nanoparticle volume fraction distributions of Ag-Ga nanofluid (*r* = 20 nm, *φ* = 0.01) in the horizontal rectangular enclosure (*A* = 2:1 and *A* = 4:1). **a** *Ra* = 1 × 10^3^, *A* = 2:1. **b** *Ra* = 1 × 10^5^, *A* = 2:1. **c** *Ra* = 1 × 10^3^, *A* = 4:1. **d** *Ra* = 1 × 10^5^, *A* = 4:1

Figure 3 presents the nanoparticle volume fraction distributions of Ag-Ga nanofluid (*r* = 20 nm, *φ* = 0.01) in the horizontal rectangular enclosure at different Rayleigh numbers (*Ra* = 1 × 10^3^ and *Ra* = 1 × 10^5^) respectively. It can be seen from Fig. 3 that the high nanoparticle volume fraction mainly distributes in the left and right sides (the center of the vortexes) of the enclosure. The velocity in the center of the vortex is smaller than that outside of vortexes. Due to the smaller velocity of nanoparticles compared with water, the nanoparticles mainly fall into the center of the two big vortexes in the left and right sides of the enclosure.

There are driving force *F*_S_ and interaction forces between particles including gravity and buoyancy force *F*_H_, drag force (Stokes force) *F*_D_, interaction potential force *F*_A_, and Brownian force *F*_B_ for nanofluid. For *Ra* = 10^5^ and *φ* = 0.01, the ranges of interaction forces between particles in enclosure with *A* = 2:1 and *A* = 4:1 are given in Tables 3 and 4 respectively. It can be seen from these two tables that the driving force *F*_S_ is the biggest force, and among the interaction forces between particles, Brownian force *F*_B_ is the biggest force, followed by the drag force *F*_D_. It can be also seen that Brownian force *F*_B_ is about two magnitudes greater than drag force *F*_D_, which is another reason for the enhancement of nanofluid in addition to the high thermal conductivity of nanofluid. In addition, it can be obtained a conclusion that the values of the driving force *F*_S_ in enclosure (*A* = 4:1) are about twice the values of *F*_S_ in enclosure (*A* = 2:1) while those of the other forces are almost the same, which causes a higher heat transfer enhancement ratio in enclosure with *A* = 4:1 compared with that in enclosure with *A* = 2:1.

**Table 3:** Ranges of driving force and interaction forces, *A* = 2:1. Ranges of driving force and interaction forces between particles in the nanofluid (*A* = 2:1, *Ra* = 10^5^, *φ* = 0.01)

Forces	*r* = 20 nm	*r* = 40 nm	*r* = 80 nm
*F*_S_	−1.2E−5 ~ 1.2E−5	−1.2E−5 ~ 1.2E−5	−1.2E−5 ~ 1.2E−5
*F*_A_	−3.2E−19 ~ −2E−20	−8E−19 ~ −5E−20	−2.8E−18 ~ −2E−19
*F*_B*x*_	−5E−13 ~ 5E−13	−5E−13 ~ 5E−13	−5E−13 ~ 5E−13
*F*_B*y*_	2E−14 ~ 2E−13	2E−14 ~ 2E−13	2E−14 ~ 2E−13
*F*_H_	−9E−19 ~ −1E−19	−7.5E−18 ~ −5E−19	−6E−17 ~ −5E−18
*F*_D*x*_	−7E−15 ~ 7E−15	−1.2E−14 ~ 1.2E−14	−2E−14 ~ 2E−14
*F*_D*y*_	−8E−15 ~ 7E−15	−1.4E−14 ~ 1.2E−14	−2E−14 ~ 2E−14

**Table 4:** Ranges of driving force and interaction forces, *A* = 4:1. Ranges of driving force and interaction forces between particles in the nanofluid (*A* = 4:1, *Ra* = 10^5^, *φ* = 0.01)

Forces	*r* = 20 nm	*r* = 40 nm	*r* = 80 nm
*F*_S_	−2.5E−5 ~ 2.5E−5	−2.5E−5 ~ 2.5E−5	−2.5E−5 ~ 2.5E−5
*F*_A_	−3.2E−19 ~ −2E−20	−8E−19 ~ −5E−20	−2.8E−18 ~ −2E−19
*F*_B*x*_	−5E−13 ~ 5E−13	−5E−13 ~ 5E−13	−5E−13 ~ 5E−13
*F*_B*y*_	2E−14 ~ 2E−13	2E−14 ~ 2E−13	2E−14 ~ 2E−13
*F*_H_	−9.5E−19 ~ −5E−20	−7.5E−18 ~ −5E−19	−6E−17 ~ −5E−18
*F*_D*x*_	−7E−15 ~ 7E−15	−1.2E−14 ~ 1.6E−14	−2.5E−14 ~ 2.5E−14
*F*_D*y*_	−8E−15 ~ 7E−15	−1.4E−14 ~ 1.2E−14	−2E−14 ~ 2E−14

**Fig. 4:**
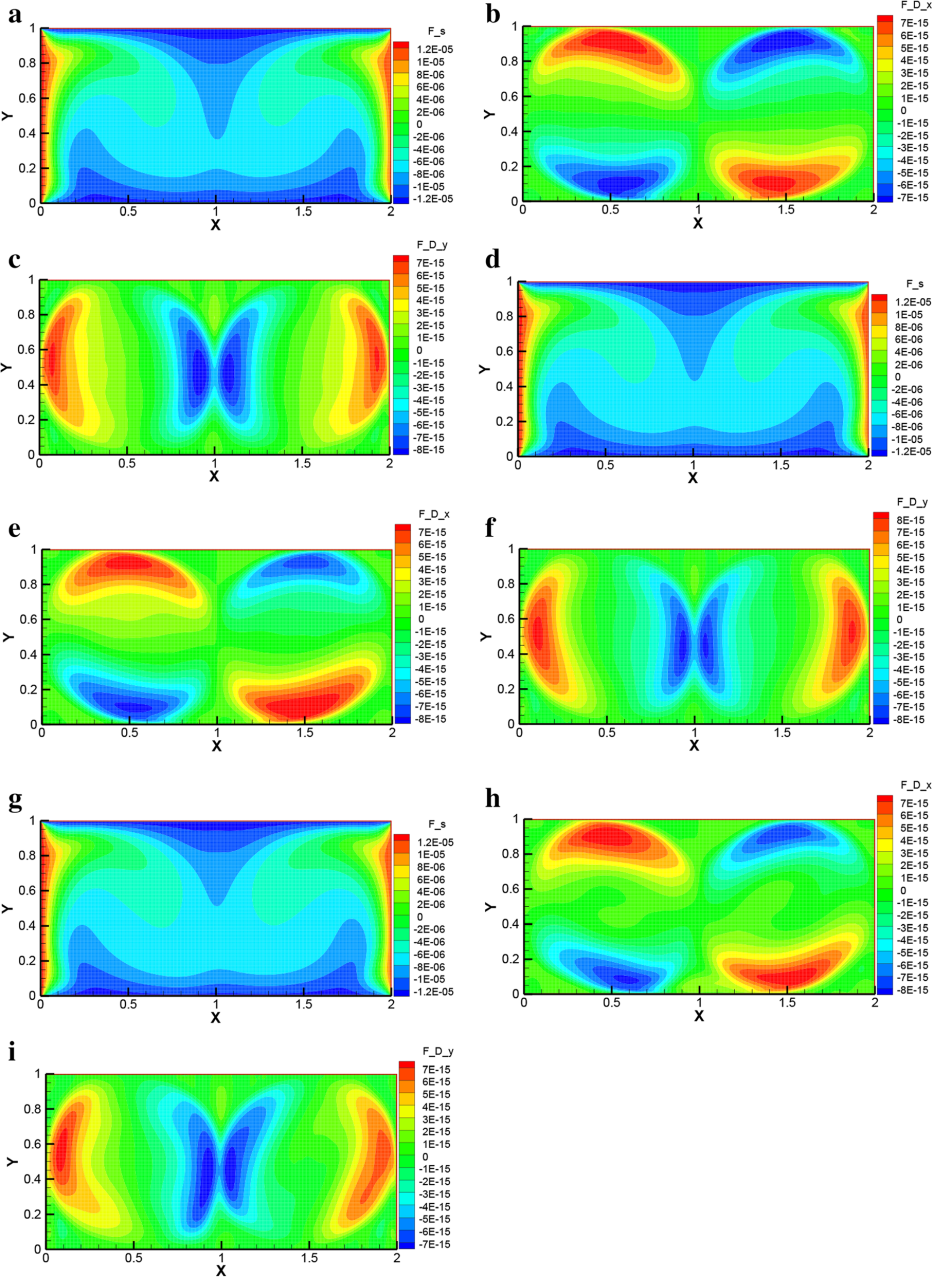
Temperature difference driving force *F*_S_ and drag force *F*_D_. Temperature difference driving force *F*_S_ and drag force *F*_D_ at *Ra* = 1 × 10^5^ (*A* = 2:1, *r* = 20 nm). **a** *F*_S_, *φ* = 0.01. **b** *F*_D*x*_, *φ* = 0.01. **c** *F*_D*y*_, *φ* = 0.01. **d** *F*_S_, *φ* = 0.03. **e** *F*_D*x*_, *φ* = 0.03. **f** *F*_D*y*_, *φ* = 0.03. **g** *F*_S_, *φ* = 0.05. **h** *F*_D*x*_, *φ* = 0.05. **i** *F*_D*y*_, *φ* = 0.05

Figure 4 shows the biggest advantageous force *F*_S_ and the biggest disadvantageous force *F*_D_ distributions at high Rayleigh number *Ra* = 1 × 10^5^ (*A* = 2:1, *r* = 20 nm). It can be seen that the driving force *F*_S_ distribution is similar to the temperature distribution. This is because the driving force *F*_S_ is the biggest force and plays a main role in the temperature distribution. The drag force *F*_D_ mainly surrounds the border of the vortex in the enclosure. This is because the velocity of nanofluid and the corresponding velocity difference between nanoparticle and gallium surrounding the border of the vortex are all bigger than that in other places.

**Fig. 5:**
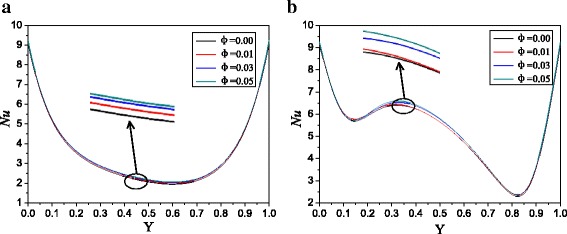
Nusselt number distributions with different volume fractions, *A* = 2:1. Nusselt number distributions of Ag-Ga nanofluid with different volume fractions along with the left hot wall, *A* = 2:1, *r* = 20 nm. **a** *Ra* = 1 × 10^3^. **b** *Ra* = 1 × 10^5^

**Fig. 6:**
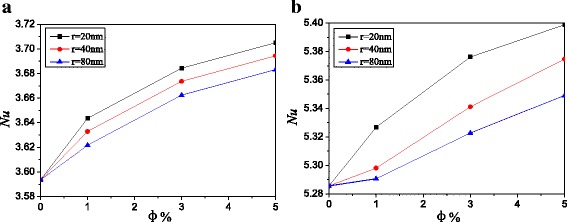
Average Nusselt numbers with different nanoparticle radiuses, *A* = 2:1. Average Nusselt numbers of Ag-Ga nanofluid with different nanoparticle radiuses along with the left hot wall, *A* = 2:1. **a** *Ra* = 10^3^. **b** *Ra* = 10^5^

Take the nanoparticle radius *r* = 20 nm and the aspect ratio of enclosure *A* = 2:1, for example, Figs. 5 and 6 give the Nusselt number distributions and average Nusselt numbers of Ag-Ga nanofluid with different nanoparticle volume fractions along with the left hot wall at different Rayleigh numbers respectively. It can be seen from Fig. 5 that Nusselt number increases with the nanoparticle volume fraction. High nanoparticle volume fraction can enhance the whole thermal conductivity of nanofluid and improve the natural convection heat transfer. It can be also seen from Fig. 5 that the Nusselt number firstly decreases with *Y* and then increases with *Y*, and there is only one Nusselt number valley at low Rayleigh number. However, there are two Nusselt number valleys at high Rayleigh number. At low Rayleigh number, there are no obvious vortexes between the two big vortexes (Fig. 2c), and the velocity in the place between the two big vortexes is small, which causes small Nusselt numbers. At high Rayleigh number, there are two small vortexes between the two big vortexes (Fig. 2d), which disturb the laminar boundary layer and enhance the Nusselt number. Hence, there are two Nusselt number valleys at high Rayleigh number, while there is only one Nusselt number valley at low Rayleigh number, and it shows a more fluctuation in the Nusselt number distributions along with the left hot wall in Fig. 5b compared with that in Fig. 5a. It can be seen from Fig. 6 that Nusselt number increases with the decrease of the nanoparticle radius. For *A* = 2:1, Ag-Ga nanofluid with the smallest nanoparticle radius (*r* = 20 nm) can enhance the heat transfer by 3.1 and 2.1% compared with water at *Ra* = 10^3^ and *Ra* = 10^5^ respectively.

**Fig. 7:**
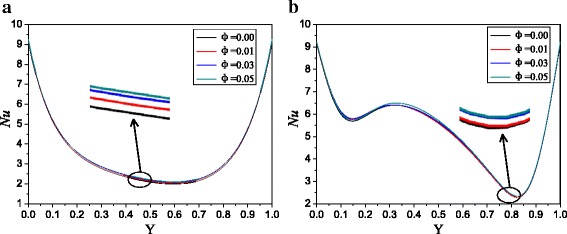
Nusselt number distributions with different volume fractions, *A =* 4:1. Nusselt number distributions of Ag-Ga nanofluid with different volume fractions along with the left hot wall, *A =* 4:1, *r* = 20 nm. **a** *Ra* = 1 × 10^3^. **b** *Ra* = 1 × 10^5^

**Fig. 8:**
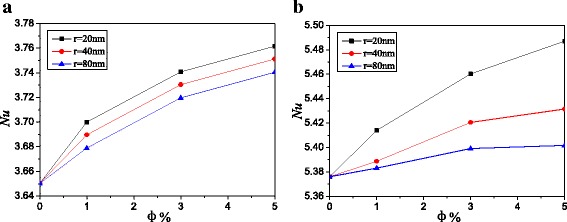
Average Nusselt numbers with different nanoparticle radiuses, *A* = 4:1. Average Nusselt numbers of Ag-Ga nanofluid with different nanoparticle radiuses along with the left hot wall, *A* = 4:1. **a** *Ra* = 10^3^. **b** *Ra* = 10^5^

The similar conclusions can be obtained from Figs. 7 and 8 compared with Figs. 5 and 6. For *A* = 4:1, Ag-Ga nanofluid with the smallest nanoparticle radius (*r* = 20 nm) can enhance the heat transfer by 3.1 and 2.1% compared with water at *Ra* = 10^3^ and *Ra* = 10^5^ respectively. The enhancement ratios of the two enclosures (*A* = 4:1 and *A* = 2:1) filled with Ag-Ga nanofluid (*r* = 20 nm) are the same.

**Fig. 9:**
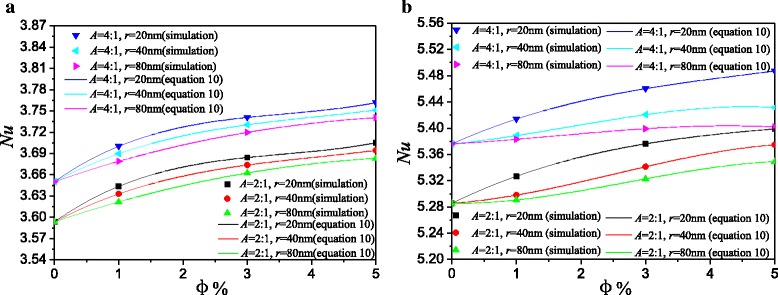
Comparison of Average Nusselt numbers between *A* = 2:1 and *A* = 4:1. Average Nusselt numbers of Ag-Ga nanofluid with different nanoparticle radiuses along with the left hot wall. **a** *Ra* = 10^3^. **b** *Ra* = 10^5^

Figure 9 shows the average Nusselt numbers of Ag-Ga nanofluid with different nanoparticle radiuses along with the left hot wall. Based on these data in Fig. 9, a mathematic correlation between average Nusselt number and nanoparticle volume fraction is given in Eq. (11). The simulation results and the results in Eq. (11) are all shown in Fig. 9. They have a good agreement with each other. It is found that the more flat horizontal rectangular enclosure (aspect ratio *A* = 4:1) has the higher Nusselt number than the less flat horizontal rectangular enclosure (aspect ratio *A* = 2:1). For every nanoparticle volume fraction, Nusselt numbers of Ag-Ga nanofluid with the smallest nanoparticle radius (*r* = 20 nm) in enclosure (*A* = 4:1) are all 1.5% higher than that in enclosure (*A* = 2:1) at low Rayleigh number *Ra* = 10^3^. Nusselt numbers of nanofluid (*r* = 20 nm) in enclosure (*A* = 4:1) are 1.6, 1.1, and 1.0% higher than that in enclosure (*A* = 2:1) for *φ* = 5%, *φ* = 3%, and *φ* = 1% at high Rayleigh number *Ra* = 10^5^ respectively. Big aspect ratio of the horizontal rectangular enclosure in this paper is advantageous to the heat transfer enhancement.

11 $$ N{u}_{\mathrm{avg}}= a+ b\varphi + c{\varphi}^2+ d{\varphi}^3 $$

where 0 ≤ *φ* ≤ 0.05 and the constants *a*, *b*, *c*, and *d* are all shown in the Table 5.

**Table 5:** The constant parameters in Eq. (11). The constant parameters in Eq. (11) at different Rayleigh numbers, aspect ratios, and radiuses

Rayleigh number	Aspect ratio	Radius	*a*	*b*	*c*	*d*
*Ra* = 10^3^	*A* = 2:1	*r* = 20 nm	3.59358	0.06429	−0.01577	0.00147
		*r* = 40 nm	3.59358	0.04796	−0.00939	7.64995E−5
		*r* = 80 nm	3.59358	0.03084	−0.00269	−2.08674E−5
	*A* = 4:1	*r* = 20 nm	3.65026	0.06397	−0.01564	0.00146
		*r* = 40 nm	3.65026	0.04806	−0.00941	7.68036E−4
		*r* = 80 nm	3.65026	0.03163	−0.00299	5.39622E−5
*Ra* = 10^5^	*A* = 2:1	*r* = 20 nm	5.28555	0.04797	−0.00721	4.30087E−4
		*r* = 40 nm	5.28555	0.00677	0.00651	−8.59108E−4
		*r* = 80 nm	5.28555	−0.00136	0.00725	−8.87629E−4
	*A* = 4:1	*r* = 20 nm	5.37596	0.04436	−0.00691	4.97021E−4
		*r* = 40 nm	5.37596	0.00938	0.00406	−7.42333E−4
		*r* = 80 nm	5.37596	0.00554	0.00195	−4.0572E−4

Table 6 shows the Nusselt number enhancement ratios for every nanoparticle radius reducing by half. It can be seen that Nusselt number enhancement ratios for every nanoparticle radius reducing by half are all about 0.3% for two enclosures (*A* = 2:1 and *A* = 4:1) at *Ra* = 10^3^. This is because the heat conduction plays a major role in the heat transfer, and the Nusselt number enhancement ratios mainly depend on the heat transfer area increment at the same nanoparticle volume fraction. The heat transfer area increment for every nanoparticle radius reducing by half are almost the same, which causes the same Nusselt number enhancement ratios at *Ra* = 10^3^. For *Ra* = 10^5^, Nusselt number enhancement ratios from the nanoparticle radius *r* = 80 nm to *r* = 40 nm for nanofluid (*φ* = 0.1%) in enclosure (*A* = 2:1 and *A* = 4:1) are smaller than those in other conditions. At high Rayleigh number *Ra* = 10^5^, the convection heat transfer plays a major role in the heat transfer, due to the low nanoparticle volume fraction (*φ =* 1%) and the small heat transfer area for *r* = 80 nm and *r* = 40 nm; the Nusselt number enhancement ratios are smaller compared with those of the others. Except above two conditions, the Nusselt number enhancement ratios at *Ra* = 10^5^ are all higher than those at *Ra* = 10^3^. High Rayleigh number can enhance the disturbance and improve the heat transfer.

**Table 6:** Nusselt number enhancement ratios. Nusselt number enhancement ratios for every nanoparticle radius reducing by half

*A*	*φ*	*Ra* = 10^3^	*Ra* = 10^3^	*Ra* = 10^5^	*Ra* = 10^5^
		$\frac{N{u}_{r=20}- N{u}_{r=40}}{N{u}_{r=40}}$	$\frac{N{u}_{r=40}- N{u}_{r=80}}{N{u}_{r=80}}$	$\frac{N{u}_{r=20}- N{u}_{r=40}}{N{u}_{r=40}}$	$\frac{N{u}_{r=40}- N{u}_{r=80}}{N{u}_{r=80}}$
2:1	1%	0.3%	0.3%	0.54%	0.14%
	3%	0.28%	0.3%	0.65%	0.35%
	5%	0.29%	0.3%	0.45%	0.48%
4:1	1%	0.29%	0.29%	0.47%	0.10%
	3%	0.28%	0.29%	0.73%	0.40%
	5%	0.28%	0.29%	1.0%	0.56%

## Conclusions

The natural convection heat transfer of Ag-Ga nanofluid with different nanoparticle radiuses in horizontal rectangle enclosures with different aspect ratios is simulated based on a two-phase lattice Boltzmann model. Some conclusions are obtained as follows:

1. Nusselt number increases with the decrease of the nanoparticle radius. The Nusselt number enhancement ratios of two enclosures (*A* = 4:1 and *A* = 2:1) filled with Ag-Ga nanofluid (*r* = 20 nm) are the same compared with those of the water at the corresponding enclosure. For both *A* = 4:1 and *A* = 2:1, Ag-Ga nanofluid with the smallest nanoparticle radius (*r* = 20 nm) can enhance the heat transfer by 3.1 and 2.1% at best compared with water at *Ra* = 10^3^ and *Ra* = 10^5^ respectively.
2. The more flat horizontal rectangular enclosure (*A* = 4:1) has the higher Nusselt number than the less flat horizontal rectangular enclosure (*A* = 2:1). Nusselt numbers of Ag-Ga nanofluid (*r* = 20 nm) in the enclosure (*A* = 4:1) are all 1.5% higher than those in enclosure (*A* = 2:1) for every nanoparticle volume fraction at *Ra* = 10^3^. For *Ra* = 10^5^, Nusselt numbers of the enclosure (*A* = 4:1) are 1.0, 1.1, and 1.6% higher than those in enclosure (*A* = 2:1) for *φ* = 1%, *φ* = 3%, and *φ* = 5% respectively.
3. Nusselt number enhancement ratios for every nanoparticle radius reducing by half at high Rayleigh number are higher than those at low Rayleigh number in most cases. For the two enclosures (*A* = 2:1 and *A* = 4:1), Nusselt number enhancement ratios for every nanoparticle radius reducing by half are all about 0.3% at *Ra* = 10^3^, and most of them are 0.35 to 1.0% at *Ra* = 10^5^.
4. The Brownian force *F*_B_ is about two magnitudes greater than the drag force *F*_D_. The value of driving force *F*_S_ in *A* = 4:1 enclosure is about twice the value of driving force *F*_S_ in *A* = 2:1 enclosure while other forces are almost the same.
